# Perceived workplace stressors and professional experiences of clinical embryologists working in Italy and Spain: a pilot qualitative study

**DOI:** 10.3389/fpubh.2025.1550715

**Published:** 2025-07-18

**Authors:** Giancarlo Astro, Monica Gatti, Mauro Costa, Arianna Cosmelli, Alessandra Alteri, Attilio Anastasi, Danilo Cimadomo, Lucia De Santis, Francesca Gioia Klinger, Emanuele Licata, Laura Sosa Fernandez, Giovanna Tomasi, Elena Vegni, Irene Cuevas Sáiz, Nicolas Prados, Valerio Pisaturo

**Affiliations:** ^1^RN, MSN, Palliative Care, Gigi Ghirotti Foundation, Genoa, Italy; ^2^Reproductive Medicine Department, International Evangelical Hospital, Genoa, Italy; ^3^Obstetrics and Gynaecology Unit, IRCCS San Raffaele Scientific Institute, Milan, Italy; ^4^Center of Physiopathology of Human Reproduction, “Del Delta” Hospital, AUSL Ferrara, Ferrara, Italy; ^5^The Italian Society of Embryology, Reproduction, Research (SIERR), Giarre, Italy; ^6^IVIRMA Global Research Alliance, Genera, Clinica Valle Giulia, Rome, Italy; ^7^Saint Camillus International, University of Health Sciences, Rome, Italy; ^8^Physiopathology of Reproduction and Andrology Unit, Sandro Pertini Hospital, Rome, Italy; ^9^Embryos Fertility Center, Battipaglia, Italy; ^10^CRA, Assisted Reproductive Center, Catania, Italy; ^11^Department of Health Sciences, University of Milan, Milan, Italy; ^12^Unidad de Medicina Reproductiva, Consorcio Hospital General Universitario de Valencia, Valencia, Spain; ^13^VIDA Recoletas, Seville, Spain; ^14^Department of Molecular Biology and Biochemical Engineering, Universidad Pablo de Olavide, Seville, Spain; ^15^The Spanish Society for the Study of Reproductive Biology (ASEBIR), Madrid, Spain; ^16^Department of Maternal and Child Health and Urological Sciences, Sapienza - University of Rome, Rome, Italy

**Keywords:** occupational well-being, workplace stressors, embryologist, medically assisted reproduction (MAR), qualitative research, thematic analysis

## Abstract

**Introduction:**

The well being of clinical embryologists remains a largely neglected aspect in the field of reproductive medicine, despite their critical role in the success of medically assisted reproduction (MAR) procedures. Embryologists manage complex tasks that require high precision and involve significant responsibilities, ranging from manual laboratory procedures to quality control and patient communication. This study aimed to investigate the perceptions of clinical embryologists regarding their occupational well being and to examine their subjective experiences within MAR centers.

**Methods:**

Qualitative interview study of 28 Italian and Spanish embryologists working at both public and private centers. Participants were recruited among the Italian Society of Embryology, Reproduction, and Research (SIERR) and the Spanish Association for the Study of Reproductive Biology (ASEBIR) members. The interviews were video-recorded and transcribed. Thematic analysis was utilized to identify the main themes and sub-themes.

**Results:**

The interviewees were concordant that excessive workload might affect their psychophysical well being, especially in view of a salary perceived as unsatisfactory with respect to the high responsibility and training involved by this job. In general, only public sector senior embryologists reported a manageable workload, proportionate to their salary. A key issue identified was the shortage of qualified embryologists, sometimes replaced with less specialized personnel. This might worsen a work environment often perceived challenging because of complex intra-team dynamics, management of interpersonal relationships, and unclear definition of the roles. A competitive and isolating atmosphere can slow down professional growth and limit positive networking. The participants emphasized the importance of patient counseling for their professional well being, advocating for a more intense interaction with the couples. Finally, an ergonomic laboratory environment, optimized equipment arrangement, access to natural light, and the inclusion of adjacent break rooms appear essential for reducing physical and mental fatigue and enhancing overall well being.

**Discussion:**

This study highlights the need to address embryologists' workload, compensation, and professional well being to improve MAR performance and patient care.

## 1 Introduction

Professional well being is defined as an integrative concept characterizing the quality of life with respect to an individual's health and work-related environmental, organizational, and psychosocial factors. Occupational stressors can negatively impact physical and mental health, job satisfaction, and performance, particularly in high-responsibility settings (1).

In the context of medically assisted reproduction (MAR), care providers are faced with a myriad of stressors from organizational challenges (e.g., intense time pressures and workload) to external factors such as patient unrealistic expectations (2). In particular, the professional figure of the embryologist is subject to several environmental stressors. Automation is minimal in the laboratories; therefore, numerous techniques are still conducted manually, and the staffing must be proportional to the volumes of MAR cycles conducted at each clinic and the increased number of very delicate procedures involved by those cycles (3, 4). These activities involve a long time at the microscope and at the computer and are associated with physical and mental fatigue. Furthermore, the high level of attention demanded by laboratory procedures encompassing complex tasks and potentially severe errors may induce additional mental stress. Other elements that contribute to this feeling include the substantial paperwork entailed to ensure efficient quality control and traceability tasks, the instances of high work intensity, and the rather unpredictable work schedule in most clinics. Communicating with patients, which also involves dealing with their adverse emotional reactions, particularly in case of bad news, represents a considerable additional stressor for MAR providers in general, although being a peculiarity for embryologists (5–7).

As for other clinical contexts, occupational stress should be kept under control to minimize a progressive worsening of professional well being, job disprivatection, increased anxiety, and behavioral disorders, such as isolation and ineffective communication within the team (8). In fact, in turn, also the quality of patient care (efficacy and safety) might suffer from the consequences of occupational stress (9). From a purely personal perspective, then, increased work absenteeism, increased “intention to leave,” and excessive turnover within operational units are all experienced by overstressed professionals (10), potentially resulting into a burnout syndrome (11), whose symptoms are physical and emotional exhaustion, apathy, and serious somatic and psycho-pathological consequences.

A few national studies investigated the occupational demands of clinical embryologists, suggesting a high risk of burnout syndrome (12, 13). Of note, in view of the reciprocal influences between patients and clinical staff, embryologists' professional well being shall not be neglected (14).

Therefore, the aim of this study was to qualitatively explore the perceived workplace stressors and professional experiences of embryologists working in Italian and Spanish MAR centers. The results are intended to serve as a foundational step for the development of a comprehensive quantitative study that will systematically assess these aspects across a broader European cohort.

## 2 Methods

### 2.1 Study design

Because of the largely unexplored topic, a qualitative analytic approach was chosen, which was based on semi-structured interviews and thematic analysis on verbatim transcribed material to allow us understanding clinical embryologists' view about their professional well being (15). Semi-structured interviews are generally used as a phenomenological approach to explore the experiences of healthcare practitioners (16). The development, conduct, and reporting of this study fulfill the COREQ criteria, which states that semi-structured interviews are suitable to identify potentially modifiable factors to enhance healthcare, in line with this study's objective (17).

### 2.2 Participants

The study was performed in collaboration with the Italian Society of Embryology, Reproduction, and Research (SIERR). Ethical Committee approval was not deemed necessary for this study as it did not involve any production, analysis or sharing of clinical or personal data. All participating professionals consented to the treatment of the information for publication purposes, and all data collected were handled with rigorous confidentiality. Privacy was ensured by the anonymization of all sensible information pertaining to the embryologists and their involvement in the study. The study was advertised on SIERR social media channels in order to recruit embryologists willing to participate. Among all members who expressed interest and were contacted in January 2023, we selected only participants who were actively working as embryologists in a MAR clinic, and the selection was made based on convenience (i.e., participants were recruited according to their availability coinciding with that of the researchers). No restrictions were applied regarding age, gender, educational background, or work experience.

All participants received an invitation letter and signed a written informed consent for audio and video recording, detailing the study's objective, participation characteristics, and methods. To obtain qualitative data from a more diverse population, the study was extended to include a cohort of Spanish embryologists. This extension was conducted in collaboration with the Spanish Association for the Study of Reproductive Biology (ASEBIR), which facilitated recruitment by sending email invitations to its members. The recruitment process followed the same methodology used for the Italian sample.

### 2.3 Procedure

In line with the framework proposed by Kallio et al. (18), the development and validation of the qualitative semi-structured interview for this study followed five key stages. Initially, we identified the prerequisites for using a semi-structured interview by clarifying the objectives of this study and assessing whether this method was the most appropriate approach for our research. Next, we conducted a comprehensive review of the literature to retrieve and apply existing knowledge relevant to the study. Following this, we formulated the preliminary semi-structured interview guide. The questions were designed to be open-ended and flexible, allowing participants to share their experiences and insights while ensuring that the key topics of the study were adequately explored.

The fourth stage involved pilot testing the preliminary interview guide with a panel of expert embryologists (AAl, LSF, GT, and VP). This step was crucial for evaluating the clarity and relevance of the questions, as well as for assessing the overall structure and flow of the interview. Finally, the interview guide was refined based on the feedback obtained during the pilot phase. The revised version was then used in the actual interviews, ensuring that the guide was both robust and effective in capturing the necessary data for the study.

**Figure 1:**
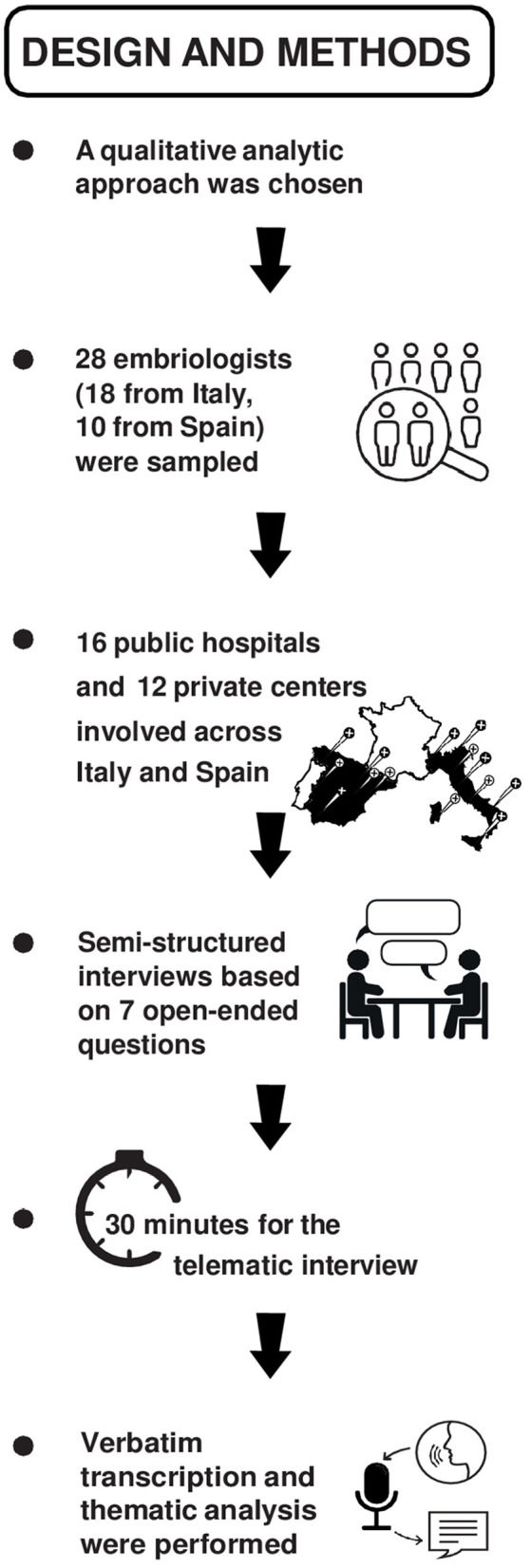
Roadmap and design of the study.

The open-ended, semi-structured interviews were conducted between January and March 2023 for the Italian sample and from July to September 2024 for Spanish sample, recorded remotely via Skype by the first author and a clinical psychotherapist. The interviews occurred on a prearranged and mutually agreed day and consisted of seven semi-structured open-ended questions, allowing the participants to freely express themselves, and facilitating in-depth exploration by the interviewer. Each interview lasted 25–45 min. The introductive question was: “Could tell me about a professional event or situation you have experienced that may have positively or negatively influenced your psychological well being?” followed by questions about their relationships with colleagues and patients, the perception of salary relatively to their workload and job responsibilities, and the ethical challenges of being a clinical embryologist, e.g., “How would you describe your working relationship with your colleagues?;” “Do you consider your salary adequate to the workload and to your job responsibility?;” “How do you handle the ethical dilemmas associated with your profession?” All interviews were transcribed verbatim. Figure 1 summarizes the workflow of the study, and the complete interview guide has been provided as Supplementary material S1.

### 2.4 Data analysis

To analyze transcribed interviews, we used the thematic analysis method proposed by Braun and Clarke (19), selected for its accessibility and flexibility. This method involves six steps: (i) becoming familiar with the data, (ii) generating initial codes, (iii) searching for themes, (iv) reviewing themes, (v) defining and naming themes, and (vi) selecting compelling extract examples.

Three authors (G.A., M.G., and A.C.) independently conducted the analysis. They continuously shared and discussed emerging themes throughout the analytical process. The initial codes were generated by three researchers through multiple readings of the transcripts. Each researcher independently identified significant textual segments related to professional experiences, stressors, relational dynamics, and coping strategies. After independent coding, discrepancies were discussed and resolved through consensus. Codes were then grouped into broader conceptual categories based on thematic similarity and relevance. This iterative process led to the identification of six overarching themes. For example, the quote “*Sometimes I feel like I'm doing the work of two people because we're understaffed”* was initially coded as “*understaffing,”* then grouped under the subtheme “*personnel shortage*,” which contributed to the development of the overarching theme “*workload and salary*.” This illustrates the inductive progression from data to theme, ensuring a grounded yet structured interpretation of participants' experiences.

The interviewing process for the Italian sample was concluded after the 18th interview as no new themes were emerging (20), indicating data saturation. For the Spanish cohort, an additional 10 interviews were conducted, which largely confirmed the thematic structure identified in the Italian sample, with only one additional theme emerging. Given the consistency of responses and the absence of novel elements in the final interviews, data saturation was considered to have been reached for the Spanish group as well.

### 2.5 Statistical analysis

Data analysis was performed with the Statistical Package for Social Sciences (SPSS) version 21.0 (SPSS Incl., USA). All continuous variables were expressed as means ± standard deviation (SD). The baseline characteristics of the two groups were compared with the Mann–Whitney *U* test and Fisher's exact test.

## 3 Results

### 3.1 Sociodemographic characteristics of participants

Twenty-eight subjects participated in the study, comprising 18 from Italy and 10 from Spain. Participants were employed in either public hospitals (57.1%, *n* = 16) or private centers (42.8%, *n* = 12). The sample included 21 women (75.0%) and seven men (25.0%). Ages ranged from 27 to 63 years, with a mean age of ~39.5 years while work experience varied from 1 to 38 years, averaging around 11.5 years. Among the participants, 25.0% had < 5 years of work experience at the time of the interview (junior embryologists), while 75.0% had more than 5 years of experience (senior embryologists). Regarding educational qualifications, 64.3% held a master's degree, and 35.7% held a PhD. No statistically significant differences were observed between the Italian and Spanish embryologists, as shown in Table 1.

**Table 1:** Baseline characteristics of Italian and Spanish embryologists.

	**Italian embryologists (*N* = 18)**	**Spanish embryologists (*N* = 10)**	***P* value**
Age (years)	42.4 ± 9.4	36.7 ± 9.9	0.065
**Sex**			
Female	14 (77.8%)	7 (70%)	0.674
Male	4 (22.2%)	3 (30%)	
**Workplace**			
Public	10 (55.6%)	6 (60%)	1.000
Private	8 (44.4%)	4 (40%)	
Work experience (years)	12.5 ± 6.79	10.6 ± 9.7	0.325
**Years of experience**			
< 5 years	4 (22.2%)	3 (30%)	0.674
≥5 years	14 (77.8%)	7 (70%)	
**Educational qualification**			
Master's degree	12 (66.7%)	6 (60%)	1.000
PhD	6 (33.3%)	4 (40%)	

Data presented as mean ± SD for continuous variables and n (%) for categorical variables.

**Figure 2:**
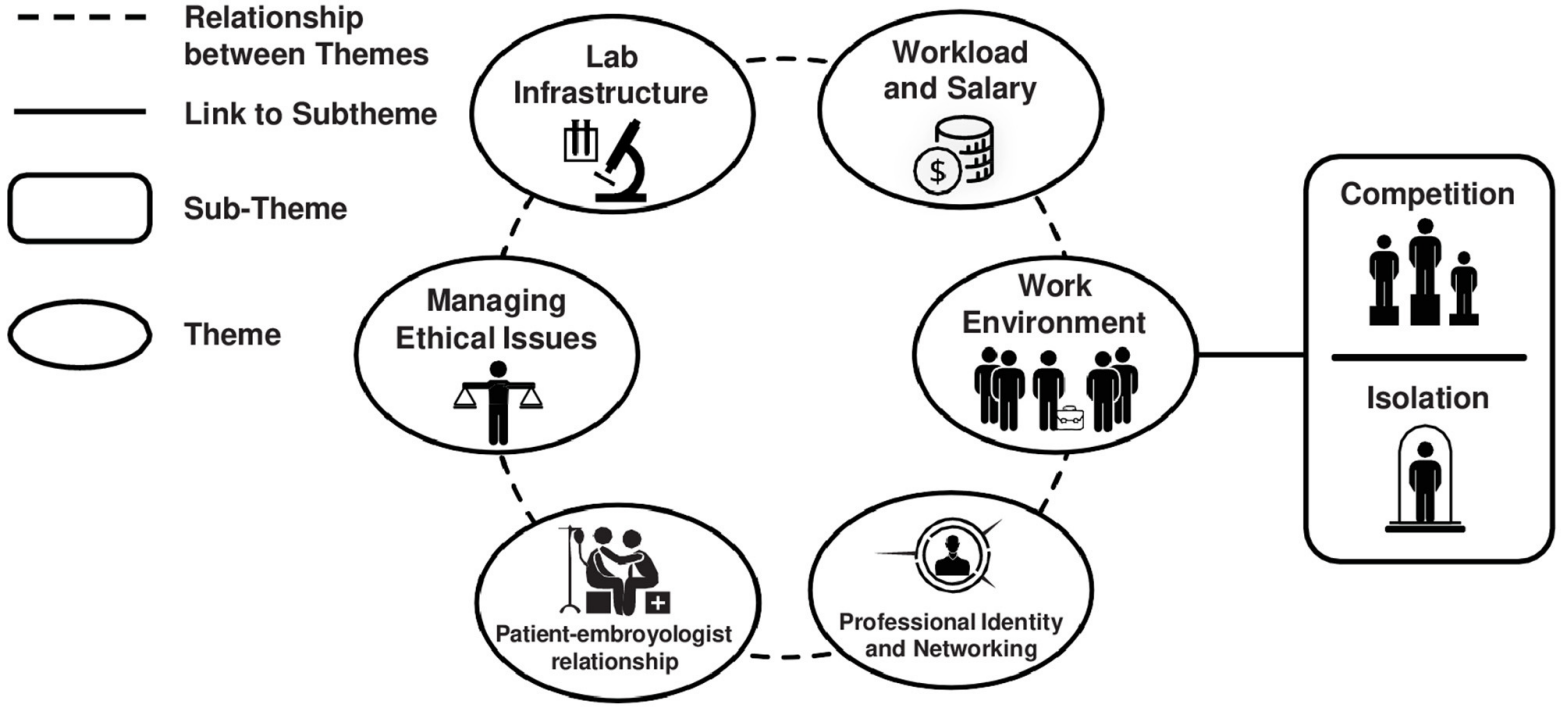
Dominant themes and subthemes extracted from textual analyses.

As a result of the process previously described, we identified six superordinate themes characterizing embryologists' experiences related to their professional well being: (i) workload and salary; (ii) work environment; (iii) professional identity and networking; (iv) patient-embryologist relationship; (v) managing ethical issues; and (vi) laboratory infrastructure. Superordinate themes and their subthemes are represented in Figure 2. The themes emerged across both the Italian and Spanish embryologists, with the exception of laboratory infrastructure, which was identified exclusively within the Spanish sample.

### 3.2 Workload and salary

The workload is perceived heterogeneously by the participants. The embryologists working at private clinics often described the workload as “very high,” or even “excessive” to the point that it may affect their psychophysical well being. The embryologists working at public hospitals also perceived their workload as “high,” but rarely defined it “excessive,” nor they reported a putative impact on psychophysical well being. The main cause identified was the shortage of embryologists, sometimes replaced by less qualified laboratory personnel, and an increasing trend in the number of MAR cycles conducted. Regardless of the professional setting, the respondents sustained that the high volumes at a center might affect the relationships with the peers in each team, and the quality of care.

“*The intense focus and attention required for extended periods and the high workload significantly impact on our psychophysical well being. Maintaining constant and intense focus throughout procedures can be mentally exhausting and potentially detrimental to well being. However, often you cannot continuously keep this control.”*

*[Senior Clinical Embryologist]*

“*In my opinion, work would be much better if only a well-defined number of Pick-Up procedures were allowed per day. This would provide emotional tranquility for us as professionals and to the patients as well. When a patient comes to the center, knowing that there are several other patients scheduled the same day, they also carry a certain emotional burden that is often overlooked*.”

*[Junior Clinical Embryologist]*

Most of the participants agreed that the salary is not adequate with respect to their responsibilities and the training required to become expert embryologists. Of note, experienced embryologists working at public hospitals, instead, perceived their compensation appropriate.

The pressure of responsibilities other than the clinical tasks (e.g., quality control or logistics), sometimes given without financial incentives, was a cause of additional stress.

“*Due to the constant attention required, the lack of tolerance for errors, and the high workload, I think my salary is not adequate, and clearly this significantly affects my professional well being! Salaries are excessively dependent on the characteristics of the center you work for and your professional category […] if you are a junior embryologist working at a private center, your salary is probably inadequate, even if you are exposed to a considerable workload and many responsibilities… I know firsthand what it's like.”*

*[Senior Clinical Embryologist]*

### 3.3 Work environment

Participants reported greater difficulty in managing intra-team relationships. A scarce definition of the responsibilities among the professional figures at each laboratory and an ineffective organization were other codes frequently associated with a negative work environment. The relationship with the gynecologist is often perceived conflictual, especially at centers where embryologists' autonomy with respect to certain work activities, such as patient counseling, is limited or constrained. The relationship with team management was often perceived as disappointing due to a poorly meritocratic environment, a limited recognition of the role of the lab in the prestige of the center, and personnel evaluation models mostly based on performance indicators (PIs) and key performance indicators (KPIs).

This superordinate theme involved one subtheme that allowed to clarify how the work environment can be perceived as competitive and isolating for our interviewees.

#### 3.3.1 Competition and isolation

The work environment is generally perceived as very competitive, mostly because of complex career advancements, the use of PIs and KPIs for operators' evaluation, and limited networking.

“*I feel that both my colleagues and I suffer from an unhealthy competition. It is as if someone is stealing work from others. I was trained in Spain for two years and this competition is felt much less there. It exists, but it is more contained because, at least in my experience, everything is better organized. With a good management, people feel positive, and their interaction improves regardless of their personalities.”*

*[Senior Clinical Embryologist]*

Participants viewed their role primarily as laboratory-based, often feeling more isolated than other professionals in terms of interaction with the patients. They often work in small teams, or even as the sole experienced embryologist, and this might lead to a sense of isolation. This lack of peer interaction for discussing procedures, also results in an increased sense of responsibility that may trigger psychological fatigue.

“*Even if the responsibility is not mine, I always feel it. When I cannot share it, it is even worse. It is not only about counseling patients, but also about making choices. Whether it is choosing a culture medium or other clinial products, or taking a given clinical decision, the fact that I cannot be reassured from my colleagues is something I truly miss.”*

*[Senior Clinical Embryologist]*.

### 3.4 Professional identity and networking

Regarding professional identity and networking, a deficit about embryologists' professional profile is generally perceived. Often, some activities are carried out by laboratory technicians or gynecologists, fostering a feeling of reduced professional autonomy. Interviewees ascribed this phenomenon to the fact that this profession is young and the legislation regulating it is still underdeveloped, with unclear directives regarding responsibilities and limits. A greater recognition of the embryologist's role is considered essential to safeguard professional well being and reduce the phenomenon of “intention to leave.” More networking among professionals is also considered key to address this challenge.

“*There should be more dialogue between clinicians and biologists, as sometimes gynecologists replace biologists in their duties, although they do not have our skills and knowledge. Everyone should cover a specific role and establish an efficient communication with the other professional figures.”*

*[Junior Clinical Embryologist]*

“*I have always requested to be involved in the decisions […], because they are purely professional choices, and I have always taken the responsibility if anything went wrong. I understand this might be difficult especially for a young embryologist. I am speaking as a 60-year-old person with almost 40 years of experience, and I imagine the situation is certainly different for young professionals. However, it is important to establish this relationship right from the beginning.”*

*[Senior Clinical Embryologist]*

Participants considered networking as a powerful tool to reduce the sense of professional isolation through experiential sharing, transforming competition into cooperation aimed at developing common projects, promoting empowerment and professional advocacy for the rights of the community of embryologists at an institutional level, and supporting professional update. The participation to scientific societies representing the Italian community of embryologists was generally promoted by the interviewees.

“*Networking is always difficult, especially with embryologists from other, often competing, clinics. It would be positive to share more with them, both professional and personal experiences, to be less closed in our laboratories and to know other professionals, combining our experiences, comparing them, creating networks, organizing educational events.”*

*[Senior Clinical Embryologist]*

### 3.5 Patient-embryologist relationship

Although the participants reported they frequently feel alienated from external relationships due to reasons intrinsic to the nature of this profession (e.g., spending a lot of time in the laboratory), there is a strong consensus that the relationship with the patients is critical for professional well being. Patients' feedback is considered important to maintain passion for the profession and many positive experiences are directly associated with moments of interactions with the couples. Being an embryologist means also communicating bad outcomes to the patients; nevertheless, this aspect of the profession is not lived with fear, it is rather felt as an opportunity to strengthen the patient-embryologist relationship. Furthermore, many respondents wish to dedicate more time to patient interactions, particularly to address their need for more comprehensive information concerning delicate IVF procedures.

“*The quality of the information given to the patients is important, especially providing them with the explanation and the reasons behind a particular event. It is easier to receive positive feedback from the couple after having accurately instructed and informed them of unpleasant news such total fertilization failure. Conversely, when explanations cannot be provided, it is normal to feel anger and disappointment.”*

*[Senior Clinical Embryologist]*

“*The emotion conveyed by the patients helps moving forward. The patients mainly interact with the clinician; however, it is also important to interact with us to make them feel that a clinic is not like a factory. This allows us giving importance to our role, not to become alienated and locked in the laboratory.”*

*[Senior Clinical Embryologist]*

### 3.6 Managing ethical issues

The multiple ethical and moral dilemmas associated with the profession were actively addressed by the participants who identified them as a possible cause of intra-personal conflict. The main dilemma is cases of treatments conducted by patients older than 45–50 years, in which the concern is associated with physical risks for both the mother and the fetus. The interviews highlighted how the coping process is based on the absolute principle that the role of the operators is “*to give a chance to a couple struggling to conceive*,” as an inalienable right independent from individual ideologies. Additionally, the confidence in the team's ability to meticulously evaluate the risks associated with older patients emerged as crucial to aid the participants in tackling their ethical dilemmas.

“*Nobody backs down from their work, but it is complicated. Ethically you think about it when you are working, and you ask yourself: ‘Is it right what I am doing?' […]. I believe we all go through it, but you do not back down because you are not alone. You know that behind you there is a medical staff who have assessed all the risks and that the couple has come to the center searching for help.”*

*[Senior Clinical Embryologist]*

The need for a specific deontological code of the embryologists often emerged in association with the theme of the ethical-moral dilemmas of the profession. Participants shared the need to develop a deontological code different from that of all biologists. The interviewees stated that they would perceive a deontological code as a significant help since embryology is an ethically complex specialty, substantially different from any other subject. A deontological code is thus, perceived as a tool capable of helping the professional understanding what is correct to perform in complex situations.

“*I think there is a true need for a deontological code, just as doctors have it and we have it for biologists. Yet, we deal with a different subject, much more specific, and often treat complex cases.”*

*[Junior Clinical Embryologist]*

### 3.7 Laboratory infrastructure

Participants from the Spanish group emphasized the importance of an ergonomic work environment, given the many hours spent daily inside their respective laboratories. They highlighted the need for a dedicated project focused on the design and construction of a purpose-built MAR lab, as many laboratories are currently housed in pre-existing hospital spaces, leading to significant logistical constraints and discomfort for healthcare professionals.

Another key aspect noted was the arrangement of equipment, which should facilitate workflow and allow for easy movement within the lab. Regarding procedural safety, participants identified access to natural light, such as through external windows, as a crucial factor in promoting well being. Interviewers reported experiencing physical and mental fatigue due to the absence of natural light.

Ergonomic chairs and appropriate support for computer monitors were also deemed necessary to prevent musculoskeletal pain among staff. Additionally, the inclusion of a break room adjacent to the lab was considered essential for ensuring professional well being, particularly during long and demanding shifts.

“*It is true that sometimes, when you are in the lab, in a place without any windows or anything like that, it can be more stressful. Also, I am not exactly sure why, but I think you just feel a little bit more tired. Now, in the new lab, we have a window, and even though we do not get direct sunlight, we can see things other than just the lab itself... I think it helps create a more relaxed work environment.”*

*[Senior Clinical Embryologist]*

## 4 Discussion

To date, only one study investigated the working experience of fertility professionals including embryologists (5), and the little knowledge of their occupational well being is mostly coming from national surveys (12, 13). We aimed at filling this gap of knowledge through a more in-depth investigation. Therefore, in this study, a qualitative analytic approach portrayed the work experiences and perceptions of 28 embryologists from Italy and Spain, identified their professional challenges and delved into their perceived occupational well being.

The themes and subthemes extracted led to the identification of sources of stress and criticalities for professionals, as well as opportunities for improvement. In both public and private settings, and especially in case of high volumes of treatments and shortage of personnel, the work of the clinical embryologists is perceived as stressful. In this regard, Alikani et al. (3) and Veiga et al. (4) emphasized how critical it is to calculate and ensure that the number of embryologists at each laboratory is properly balanced with the number of procedures they must conduct (3, 4).

The limited automation in the IVF clinics implies that this job is still nowadays very manual, and excessive workloads might affect embryologists' psychophysical well being, in particular if the relationship with their co-workers and the management of the team is perceived as sub-optimal. This job involves large responsibilities, which might intensify stress in case of high workload, poor management of the human resources, unhealthy work environment, and limited technological aid in the daily activities. The high level of concentration required for all the manual steps involved by IVF procedures, their traceability and the related paperwork, in turn limits the time dedicated to communication within the team, increases the risk of misunderstandings, and involves higher probability of relationships deterioration among co-workers as suggested by Lake et al. (9) and supported by O'Connor et al. (8). In addition, MAR procedures have evolved to include several complex techniques, which are associated with various risks of errors and failure opportunities, especially in laboratories (21). Although total eradication of error in the laboratory may be impossible, several innovative risk management programs and technologies can minimize these risks (22). In this context, minimizing or removing these stress-inducing factors can help reduce the likelihood of human errors. The interviewees were concordant that automation of witnessing steps for gamete and embryo tracking is beneficial, and that more technological advancements in general in the IVF laboratories are desirable. This finding is consistent with previous research, which has shown that the introduction of electronic witnessing systems has already demonstrated positive impacts on embryologists' well being, enhancing their confidence and reducing stress, which in turn leads to improved job satisfaction and mental health (23). From a purely financial perspective, there was a general perception that the salary is inadequate with respect to the responsibilities and the workload. Moreover, it should be noted that some private MAR centers may be owned or operated by equity-backed organizations, which often emphasize productivity, efficiency, and financial performance. This type of ownership structure could contribute to increased pressure on embryologists, influencing workload expectations, organizational policies, and job satisfaction. These are all aspects that should be carefully addressed, as they are risk factors for burnout syndrome (11), lower clinical performance, and work absenteeism due to higher “intention to leave” phenomena (10). In this context, a work environment with positive relationships between colleagues and peers is essential to keep good levels of professional well being. Instead, communication with co-workers was perceived as even more complicated than counseling patients, and feelings of competition and isolation might be issued from these dysfunctional relationships, as previously suggested also by Chari et al. (1). If embryologists' performance is assessed mostly based on clinical PIs and KPIs (24), this might further enhance feelings of competition and isolation. It is not surprising that many embryologists reported seeking psychological support, confirming the need for mental health professionals in the fertility staff (5). In general, better management of the daily schedule to equally distribute the number of treatments throughout the working week might ease the logistics of the laboratory and, thus, improve the inter-personal relationships in it. Artificial intelligence tools might support this task in the coming years (25) and help the clinics to cope with the constantly increasing request for IVF, which at present already contributes for 2%−3% of the babies born every year in Italy and 9%−11% in Spain (26).

The Italian and Spanish embryologists interviewed reported that the current legislative background impacts their feeling of professional identity. Specifically, the role of the embryologist in Italy and in Spain is poorly defined, and many areas of competence are therefore, shared with other healthcare professionals, causing frictions in the team. This feeling is not peculiar of Italians. In 2020, an expert meeting was organized by SIERR in Rome to address the professional status and the educational paths to become Clinical Embryologists, further expanded through a collaboration with ESHRE. Many European countries do not formally recognize Clinical Embryology as a profession, and embryologists are often registered under other healthcare professions (e.g., biologists), leading to a significant disparity in educational and training programs as well as in competence monitoring policies (27).

Moreover, in many IVF centers, the laboratory staff, including embryologists, often remain unknown to other members of the healthcare team, as well as to patients. This lack of exposure and recognition could significantly impact how embryologists perceive their professional value and identity in relation to their employers and patients. In this regard, networking is generally perceived as a very effective tool to address this issue. Increased interaction between senior and junior embryologists, both in the public and private contexts, would facilitate the advancement of projects aimed at professional empowerment and promote continuous education. Moreover, networking can be a tool for raising awareness within the embryology community about the importance of occupational well being and would enable the generation of proposals aimed at improving current professional conditions. Finally, the literature supports the association between networking and enhanced quality and safety of care provided (28). Also, interactions with patients often represent the most fulfilling professional memories and the primary source of positive feedback. Indeed, according to the “The Integrated Approach for Fertility Care” model (14), promoting embryologists-patients relationship could improve both professional well being and couples' coping processes.

Another important topic that emerged from the interviews is the need for a specific deontological code for Italian and Spanish embryologists.

Ethical concerns are not new in the field of MAR, and clinical embryologists are often required to make complex decisions regarding patient selection and treatment eligibility. Deontological ethics in the context of medically assisted reproduction is a moral framework that evaluates actions based on universal principles such as autonomy, beneficence, and justice. It emphasizes the ethical responsibilities of clinicians and embryologists toward patients and gametes, regardless of outcomes, and raises fundamental questions, such as whether embryos should be regarded as persons or property. Establishing a profession-specific deontological code may support embryologists in navigating these ethically charged situations, helping them manage the emotional burden and stress associated with the high volume of delicate procedures they perform daily, particularly given the increasing number of older patients seeking IVF treatment (6).

Finally, laboratory infrastructure plays a significant role in the occupational well being of embryologists. This theme emerged exclusively in the Spanish group, where participants highlighted the challenges posed by laboratories often being housed in repurposed architectural spaces without substantial infrastructural modifications. These limitations in space management can negatively impact embryologists' daily work due to logistical constraints. In addition, Spanish embryologists emphasized the importance of access to natural light, reporting both physical and mental fatigue when natural light was absent from the laboratory environment. This finding aligns with the study by Pisaturo et al. (29), which highlighted the critical role of adequate lighting in ART laboratories. While excessive light exposure may pose potential risks to gametes and embryos, the absence of proper lighting can lead to procedural errors and negatively impact staff well being (29).

The strengths of this study lie in its methodology, that allowed a deep exploration of topics scarcely investigated previously. However, this study shows several limitations. First, despite including embryologists from two different countries, it remains constrained by not extending the analysis to a broader range of European countries. Furthermore, although the sample size is consistent with the chosen methodology, this study summarizes the views of only 3% of Italian embryologists working in 7% of Italian MAR centers and 1% of Spanish embryologists working in 3% of Spanish MAR centers. Then, the small number of participants prevents definitive conclusions regarding potential differences or similarities between the two groups. Additionally, there is a potential for selection bias, as it is plausible that those who participated were more closely connected to the organizing societies (SIERR and ASEBIR), possibly sharing similar perspectives. Moreover, the present study did not account for the territorial distribution of participants within each country. Given the known regional differences in income levels, work conditions, and healthcare infrastructure, particularly relevant in both Italy and Spain, future research should consider analyzing the geographical representation of embryologists to better understand how local contexts may influence occupational well being. In addition, although the perceived shortage of embryologists emerged as a key theme, we did not quantitatively assess staffing disparities between public and private centers. Including workload-to-staff ratios or reference data from professional societies would have strengthened the analysis, but such metrics were not systematically available at the time of the study. This aspect should be further explored in future research. Finally, we did not systematically collect data regarding the number of ART cycles performed at each facility or the number of embryologists employed per center. These variables are crucial to contextualize perceived workload and allow more precise interpretation of differences across facilities. To better understand how staffing influences occupational well being, future research should aim to collect more specific data on the numerical ratio between the number of embryologists and the number of patients or treatment cycles managed within a given period. This information would allow for a clearer assessment of how workload is distributed in MAR units and help identify thresholds beyond which psychological strain, job dissatisfaction, or burnout may increase. Linking these staffing ratios to self-reported well being outcomes could support the development of evidence-based staffing recommendations and ensure more sustainable working conditions across both public and private reproductive medicine centers. Future research will adopt a quantitative survey-based approach to validate and expand upon the present findings, enabling broader generalizability and more detailed analysis across diverse clinical settings.

New challenges and potential improvements are expected in the coming years with the advent of automation and artificial intelligence. If at present, the manual work in IVF is irreplaceable, future tools could substantially reduce human workload in the laboratory. Consequently, the role of the embryologist may be redefined, transitioning from being focused on technical skills to more managerial, counseling and scientific duties (30). With the incorporation of advanced tools and techniques, the workload and stress associated with intricate procedures are poised to reduce, leading to improved job satisfaction and mental health. Furthermore, as the field becomes more interdisciplinary, embryologists will benefit from increased collaboration and support, fostering a more sustainable and fulfilling work environment. This evolution, while focusing on technological progress, also promises a more nurturing and supportive professional landscape for embryologists, ultimately benefiting both the practitioners and the patients.

In light of the organizational and ethical challenges identified, practical interventions should be considered to improve working conditions and professional satisfaction. Standardizing the professional roles and responsibilities of embryologists may reduce confusion and role overlap within clinical teams, enhancing collaboration and communication. Additionally, the development of a dedicated deontological code could offer a shared ethical framework, helping professionals manage morally sensitive situations and reinforcing their sense of identity and accountability within the healthcare system.

## 5 Conclusions

National health policies should focus on the relationship between workload, salary compensation, and professional well being, and therefore, consider the beneficial effects on the quality of care that would result from a greater recognition of the embryologists' professional role. Embryology societies, as representatives of this community, should promote awareness about professional well being within MAR centers, invest in networking as a tool for professional empowerment, and assess the need to develop a specific deontological code. Ensuring embryologists' well being is not merely beneficial for the embryologists themselves but it is also critical for the quality of care and the clinical performance.

## Data Availability

The raw data supporting the conclusions of this article will be made available by the authors, without undue reservation.
