# Supplementary material for: Perceived workplace stressors and professional experiences of clinical embryologists working in Italy and Spain: a pilot qualitative study
Source: Front Public Health. 2025 Jul 18;13:1550715. doi: 10.3389/fpubh.2025.1550715 (PMC12313716; doi:10.3389/fpubh.2025.1550715)
Supplement: Supplementary file 1 [file Table_1.docx]

**Semi-structured Interview Questions:**

1. **Do you consider the workload in your profession—defined as the combination of the complexity of techniques you perform daily, the volume of tasks, and the level of responsibility inherent in your role—excessive?**
   - **Follow-up question:** Do you find that your salary is commensurate with the work you perform?
2. **How would you describe the professional relationships you have established within your team? How do you experience teamwork with your colleagues?**
   - **Follow-up question:** Do you perceive any friction within the team? If so, what do you think are the underlying causes?
3. **How do you manage the interactions your profession fosters with patients, and what emotions do these interactions evoke?**
   - **Follow-up question:** Have you ever had to deliver bad news to a patient? If so, how did this experience affect you, and how did you cope with it?
4. **Your profession frequently encounters ethically debated issues, such as the age of patients undergoing procedures, their potentially compromised physical conditions, or heterologous fertilization.**
   - **Follow-up question:** Do you believe these ethical issues impact your work-related well-being? If so, could you elaborate?
5. **Do you think the community of embryologists in Italy would benefit from a specific Code of Ethics for the profession?**
6. **Could you describe an event or professional situation that has significantly influenced your psychological well-being, either positively or negatively?**
7. **What advice would you give to the community of embryologists to improve the organizational well-being of the profession?**
